# SNMMI/ACR/ASNC/SCMR joint credentialing statement for cardiac PET/MRI

**DOI:** 10.1186/s12968-022-00867-z

**Published:** 2022-07-19

**Authors:** Terrence D. Ruddy, Mouaz Al-Mallah, James A. Arrighi, John P. Bois, David A. Bluemke, Marcelo F. Di Carli, Vasken Dilsizian, Robert J. Gropler, Hossein Jadvar, Saurabh Malhotra, Matthieu Pelletier-Galarneau, Thomas H. Schindler, Pamela K. Woodard, Panithaya Chareonthaitawee

**Affiliations:** 1 University of Ottawa Heart Institute, Ottawa, ON Canada; 2 Houston Methodist Hospital, Houston, Tx USA; 3 Warren Alpert Medical School of Brown University, Providence, RI USA; 4 Mayo Clinic, 200 First Street SW, Rochester, MN USA; 5 University of Wisconsin-Madison, Madison, WI USA; 6 Brigham and Women’s Hospital, Boston, MA USA; 7 University of Maryland School of Medicine, Baltimore, MD USA; 8 Washington University School of Medicine, St. Louis, MO USA; 9 University of Southern California, Los Angeles, CA USA; 10 Rush Medical College, Chicago, IL USA; 11 Institut Cardiologie de Montréal, Montréal, QC Canada

## Background

Founded in 1951, the Joint Commission, formally known as the Joint Commission on Accreditation of Healthcare Organizations and, previous to that, the Joint Commission on Accreditation of Hospitals, is a U.S.-based nonprofit organization that accredits more than 20,000 health-care organizations and programs in the United States. The Joint Commission requires that there be a credentialing system for delineating and granting privileges to every hospital physician. The Joint Commission does not specify the qualifications. Privileges are generally practice-specific and are not usually transferable from hospital to hospital. The granting of clinical privileges cannot and should not depend on a single criterion such as board certification or membership in a particular specialty society; other options, such as documented evidence of requisite training, relevant experience, judgment skills, and demonstrated current competence, should be available. It is the final responsibility of the hospital medical staff and hospital governing board to ensure that a physician meets a reasonable standard of competency.

Positron emission tomography (PET)/magnetic resonance imaging (MRI) is an emerging complex hybrid imaging modality recently introduced into clinical practice. In June 2013, the American College of Radiology (ACR) and the Society of Nuclear Medicine and Molecular Imaging (SNMMI) charged a joint task force with developing a credentialing statement for physicians responsible for the oversight and interpretation of PET/MRI examinations. In conjunction with the Cardiovascular Council of the SNMMI, the task force has prepared this joint statement related to cardiac PET/MR imaging as the third in a planned series of credentialing documents covering all organ systems and clinical applications. The first on PET/MRI of the brain was published in 2015.

This joint statement is intended to guide credentialing bodies that privilege physicians to oversee, supervise, and interpret cardiac PET/MRI for patient care in the United States.

## Definitions

For the purposes of this statement, the following definitions apply:

MRI: A medical imaging technology that uses high-strength magnetic fields to create high contrast tomographic images. In cardiovascular imaging, MRI techniques delineate anatomy of the heart and great vessels. For cardiac imaging, special MRI sequences with and without contrast enhancement and cardiac and respiratory gating also provide soft-tissue characterization, accurate measurements of cardiac chamber size and global and regional function, valvular morphology, and physiologic parameters.

PET: A medical imaging technology that uses positron-emitting radiopharmaceuticals. The PET scanner detects high-energy annihilation photon pairs and extrapolates the location of the original positron-emitting atoms to create tomographic images of their biodistribution within the body. In cardiac imaging, this provides an assessment of the physiologic and functional processes of the heart.

PET/MRI: A complex hybrid medical imaging technology that incorporates PET and MRI into a single device. Such an imaging system allows either sequential acquisition (i.e., tandem back-to-back design) or simultaneous acquisition (i.e., a PET insert in an MRI gantry or an integrated PET/MRI design). In either hybrid system, the goal is to combine PET and MRI data for exact co-registration. For the purposes of this document, a PET/MRI examination is defined as a full diagnostic PET examination and a full diagnostic MRI examination.

Radiopharmaceutical: A radioactive compound administered to patients for use in diagnosis and therapy.

## Applications for cardiac PET/MRI

The following are examples of potential clinical or research applications for PET/MR imaging of the heart [3–7]: rest and/or stress myocardial perfusion, myocardial viability including rest and/or stress perfusion when indicated, inflammatory processes such as sarcoidosis and myocarditis, myocardial masses including non-neoplastic and neoplastic (both primary and metastatic), infiltrative processes such as amyloidosis, hereditary disorders such as hypertrophic cardiomyopathy and Anderson-Fabry disease, cardiac valvular disorders and emerging areas of research including tissue regeneration after stem cell therapy, coronary artery plaque imaging and assessment of sympathetic reinnervation after cardiac transplant.

## Responsibilities of physicians

The physician or physicians supervising the examination must be responsible for all aspects of the cardiac PET/MR imaging examination, such as reviewing indications for the examination, prescribing the PET radiopharmaceutical and its activity, specifying the MRI pulse sequences to be performed, prescribing the use and dose of MRI contrast agents, confirming the quality of the images appropriate for interpretation, interpreting the images, generating final reports, and ensuring patient and personnel safety. A prescriber of radiopharmaceuticals must either be or work under a U.S. Nuclear Regulatory Commission authorized user of radioactive materials and comply with state regulations, or hold the Canadian equivalent authorization. These responsibilities may be distributed by expertise to two physicians each credentialed, respectively, in MRI and PET only.

With regard to supervision and interpretation of cardiac PET/MRI, physicians may best participate in their practice according to their special interests and qualifications in nuclear medicine and molecular imaging, nuclear cardiology, multimodality cardiac imaging, or related practice paradigms. It is recognized that variations in the local regulatory requirements for practices and individual-physician qualifications may of necessity dictate site-specific practice patterns. For example, in certain clinical settings, it may be beneficial for 2 physicians to interpret cardiac PET/MRI examinations together, with one primarily interpreting the PET component and other primarily interpreting the MRI component. Nevertheless, the interpreters should meet the credentialing criteria for the modality for which they are providing a primary interpretation. The 2 physicians must reach consensus regarding the final interpretation of the combined PET/MRI findings to issue a joint PET/MRI report (1 billing physician of record) or 2 separate correlative PET/MRI reports. Simple numeric criteria are not an optimal measure of competency. Documentation of competency by the use of objective, outcome-based tools related to clinical experience is preferable. The criteria detailed in Table 1 may be used for guidance.

**Table 1:** Initial and Continuing Competency Criteria for Credentialing Physicians to Independently Practice Cardiac PET/MRI

Number of interpretations (training)	Didactic requirement	Number of interpretations (continuing)	CME/MOC
100 cardiac PET examinations (applicant must be physically present and perform initial interpretation of 50 of these)*; Board certification**or equivalent; radiation safety training for NRC 35.290 imaging and localization studies (or equivalent)	20 h of didactic education is recommended	100 cardiac PET studies within prior 3 years***	Fulfilling MOC; or 15 h CME related to cardiac PET in prior 3 years
100 cardiac MRI examinations (applicant must be physically present and perform initial interpretation of 50 of these)*; Board certification **or equivalent	20 h of didactic education is recommended	100 cardiac MRI within prior 3 years***	Fulfilling MOC; or 15 h CME related to cardiac MRI in prior 3 years

An alternative and acceptable method of performance of the cardiac PET/MRI examination is supervision and interpretation by two physicians, each credentialed individually in either cardiac PET or cardiac MRI

^*^For programs with hybrid cardiac PET/MRI, combined cardiac PET/MRI examinations may count for both cardiac PET and cardiac MRI examinations. “Physical presence” implies involvement on acquisition and processing of studies

^**^ American Board of Radiology (ABR), American Board of Nuclear Medicine (ABNM), Certification Board of Nuclear Cardiology (CBNC) or the Certification Board of Cardiovascular Magnetic Resonance (CBCMR), or the equivalent in Canada

^***^For those pursuing credentials in pediatric cardiac PET/MRI, given the relatively low number of pediatric PET and MRI studies, an exception should be made that would allow for the continuing training to be more than 3 years, in order to achieve the competency criteria. A timeframe of 5 years may be reasonable, but in certain specialized clinical settings where the practice is limited only to pediatrics, the proposed criteria may be waived

The recommendations for PET/MRI practice set forth in this document have been developed in consideration of the complex nature of this emerging hybrid modality and with reference to published practice parameters, procedure guidelines, and credentialing statements.

The practice of cardiac PET/MRI requires a solid knowledge base of PET techniques and MRI techniques. Although the goal is mastery of simultaneously acquired cardiac PET/MRI examinations, the physician’s education, training, and experience should encompass dedicated cardiac PET, cardiac PET/computed tomography (CT), and dedicated cardiac MRI. Fundamentals include familiarity with technical parameters (protocols, contrast agents, sequences, processing) as well as biologic and clinical parameters (anatomy, physiology, normal variants, disease states).

It is recognized that both general radiology and cardiovascular training programs vary widely in their ability to provide experience in cardiac PET or cardiac MRI. Thus, whether a physician comes from a radiology, nuclear medicine, or cardiology training background, gaps in experience may be present after formal training. This writing committee therefore recommends that the credentialing standards for cardiac PET/MRI be the same irrespective of the primary specialty of the physician (radiology, nuclear medicine, or cardiology). This writing committee also recommends that the cardiology or radiology trainee wishing to gain competency in independent interpretation of cardiac PET/MRI have concurrent or prior training in cardiac PET and cardiac MRI as outlined in Table 1.

For those with radiology or nuclear medicine as a primary specialty wishing to interpret cardiac PET/MRI in clinical practice, this writing committee recommends that in addition to the recommended initial training and continuing requirements, the radiologists or nuclear medicine specialist also holds and maintains certification by the American Board of Radiology or the American Board of Nuclear Medicine, respectively, or the equivalent in Canada. For those with cardiology as a primary specialty wishing to interpret cardiac PET/MRI in clinical practice, this writing committee recommends that in addition to the recommended initial training and continuing requirements, that the cardiologist also hold a certificate by either the Certification Board of Nuclear Cardiology (CBNC) or the Certification Board of Cardiovascular Magnetic Resonance (CBCMR), or the equivalent in Canada.

The training in cardiac PET/MRI should be based on acquisition of competencies, and not number of procedures alone. Training must also account for baseline differences in knowledge; trainees from a cardiology-based background, for example, may begin with a more advanced understanding of cardiac anatomy and physiology, whereas those from a radiology background may begin with a more comprehensive understanding of instrumentation.

The recommendations set forth include, first, a first-hand review and supervised interpretation of hybrid cardiac PET/MRI or separately acquired PET and MRI examinations, recognizing that hybrid cardiac PET/MRI training experiences are currently unlikely to be widely available, and second, didactic educational hours in the basic science and clinical applications of each modality.

## Qualifications of physicians responsible for cardiac PET/MRI—initial competency

All physicians assuming responsibility for independent practice of cardiac PET/MRI should meet the categories for initial competency outlined in Table 1. This table defines the number and time-based criteria that are generally required to ensure sufficient exposure to the range, volume, and diversity of clinical experience necessary for competency. Ultimately, determination of whether an individual has the requisite knowledge and skill for independent practice of cardiac PET/MRI should be based on a formal assessment of competency. Those considering these thresholds should bear in mind that numbers of procedures are proxies for acquiring the technical proficiency and analytic skills essential for clinical mastery of an imaging technique. An alternative and acceptable method of performance of the cardiac PET/MRI examination is supervision and interpretation by two physicians, each credentialed individually in either cardiac PET or cardiac MRI, respectively.

### Clinical experience/supervised interpretations

Cases must include broad representation of the types of cardiac examinations, indications, available radiopharmaceuticals, and contrast agents that are encountered typically in clinical practice. For trainees (residents or fellows) who are currently in training programs, documented cases with direct participation of the trainee and appropriate supervision by attending physicians experienced in cardiac PET/MRI is preferred. Cases presented in specifically designed society or organization approved case-based conferences or courses, live or online, are acceptable. Of note, hybrid cardiac PET/MRI cases may be counted in each appropriate category to fulfill the recommendations.

### Didactic education hours

Didactic education should supplement case-based conferences used to achieve the number of supervised interpretations and should include physics relevant to cardiac PET and cardiac MRI, use of MRI contrast agents, MRI safety, radiation safety of PET radiopharmaceuticals, and issues relevant to hybrid cardiac PET/MRI.

### Training for authorized use of radiopharmaceuticals for imaging and localization studies

Training for physicians who intend to practice in the United States must satisfy Nuclear Regulatory Commission requirements for training in imaging and localization studies (10 CFR 35.290).

## Qualifications of physicians responsible for cardiac PET/MRI—continuing competency

Physicians assuming responsibility for independent practice of cardiac PET/MRI should meet competency in both cardiac PET and cardiac MRI as outlined in Table 1. An alternative and acceptable method of interpretation of the cardiac PET/MRI examination is interpretation by two individuals, each with competency in one of the two complementary modalities.

### Clinical experience/supervised interpretations

Cases must include broad representation of the types of examinations, indications, available radiopharmaceuticals, and contrast agents that are encountered typically in clinical practice. Direct participation in clinical cases should include involvement in all aspects of examination. Objective documentation of direct participation should be kept by each physician. Cases presented in specifically designed society or organization approved case-based conferences or courses, live or online, may be used to supplement ongoing experience, particularly for unusual cases that are typically encountered infrequently in clinical practice. Of note, hybrid cardiac PET/MRI cases may be counted in each appropriate category to fulfill the recommendations.

### Continued medical education and/or maintenance of certification

Evidence of ongoing commitment to lifelong learning and, if relevant, maintenance of certification in imaging, is required. A guideline for a typical number of CME hours in cardiac PET/MRI is indicated in Table 1.

## Approval

This credentialing statement was approved by the ACR Executive Committee of the Board of Chancellors on March 3, 2021, the Society for Cardiovascular Magnetic Resonance (SCMR) in February 2021, the American Heart Association (AHA) on October 27, 2021, the American Society of Nuclear Cardiology (ASNC) Board of Directors on November 30, 2021, and the SNMMI Board of Directors on January 28, 2022.

## Joint task force participants

### Representatives of Societies

ACR: Pamela K. Woodard, David Bluemke.

AHA: John Bois, Robert Gropler.

ASNC: Mouaz Al-Mallah.

SNMMI: Panithaya Chareonthaitawee (co-chair), James A. Arrighi, Marcelo F. Di Carli, Vasken Dilsizian, Hossein Jadvar, Saurabh Malhotra, Mathieu Pelletier-Galarneau, Terrence D. Ruddy (co-chair), Thomas H. Schindler.

SCMR: David A. Bluemke.

## Data Availability

No datasets were used in this manuscript.

## References

[1] Jadvar H, Subramaniam RM, Berman CG (2015). American College of Radiology and Society of Nuclear Medicine and Molecular Imaging joint credentialing statement for PET/MR imaging: Brain. J Nucl Med.

[2] Subramamiam RM, Jadvar H, Colletti PM (2017). ACR and SNMMI Joint Credentialling statement for PET/MRI of the Body. J Nucl Med.

[3] Lau JM, LaForest R, Nensa F (2017). Cardiac Applications of PET/MR Imaging. Magn Reson Imaging Clin N Am.

[4] Bergquist PJ, Chung MS, Jones A (2017). Cardiac Applications of PET-MR. Curr Cardiol Rep.

[5] Rischpler C, Nekolla SG, Dregely I (2013). Hybrid PET/MR Imaging of the heart: potential, initial experiences, and future prospects. J Nucl Med.

[6] Beitzke D, Rasul S, Lassen ML (2020). Assessment of Myocardial Viability in Ischemic Heart Disease by PET/MRI: Comparison of Left Ventricular Perfusion Hibernation and Scar Burden. Acad Radiol.

[7] Nensa F, Bamberg F, Rischpler C (2018). Hybrid cardiac imaging using PET/MRI: a joint position statement by the European Society of Cardiovascular Radiology (ESCR) and the European Association of Nuclear Medicine (EANM). Eur Radiol.

[8] Kim RJ, Simonetti OP, Westwood M (2018). Guidelines for training in cardiovascular magnetic resonance (CMR). J Cardiovasc Magn Reson.

[9] Vasken D, James AA, Rose SC, Todd DM, Allen JS, Udelson JE (2015). COCATS 4 Task Force 6: Training in Nuclear Cardiology. J Am Coll Cardiol..

[10] Christopher MK, Gregory H, Raymond YK, Matthew WM, Subha VR, Parker W (2015). COCATS 4 Task Force 8: Training in Cardiovascular Magnetic Resonance Imaging. J Am Coll Cardiol.

[11] PET Accreditation Program Requirements (ACR). https://www.acraccreditation.org/-/media/ACRAccreditation/Documents/NucMed-PET/PETRequirements.pdf?la=en

